# CardioAtlas: deciphering the single-cell transcriptome landscape in cardiovascular tissues and diseases

**DOI:** 10.1186/s40364-024-00696-5

**Published:** 2024-11-28

**Authors:** Tiantongfei Jiang, Xiaoyan Jin, Yueying Gao, Weiwei Zhou, Jinyang Yu, Yongsheng Li, Juan Xu, Benzhi Cai

**Affiliations:** 1 College of Bioinformatics Science and Technology, Harbin Medical University, Harbin, Heilongjiang 150081 China; 2 Department of Pharmacy, The Second Affiliated Hospital, Department of Pharmacology, College of Pharmacy, (The Key Laboratory of Cardiovascular Medicine Research, Ministry of Education), Harbin Medical University, Harbin, 150081 China; 3 School of Interdisciplinary Medicine and Engineering, Harbin Medical University, Harbin, 150081 China; 4 Hainan Provincial Key Laboratory for Human Reproductive Medicine and Genetic Research, Department of Reproductive Medicine, Hainan Provincial Clinical Research Center for Thalassemia, Key Laboratory of Reproductive Health Diseases Research and Translation, College of Biomedical Information and Engineering, Hainan Medical University, Ministry of Education, The First Affiliated Hospital of Hainan Medical University, Haikou, 571199 China

## Abstract

Increasing scRNA-seq data in cardiovascular research have substantially improved our knowledge on the development of the cardiovascular system and the mechanisms underlying cardiovascular diseases. However, the single-cell transcriptome datasets were dispersed in literature and no resource for cardiovascular systems and diseases. Here, we constructed an organized resource CardioAtlas, which provides comprehensive analysis results for > 1,929,000 cells in 27 human data sets and > 1,088,000 cells in 39 mouse data sets. Through large-scale literature retrieval and manual annotation, we constructed 12 and 15 scRNA-seq reference atlas for common human and mouse cardiovascular systems and diseases, covering 43 and 39 cell types. In particular, CardioAtlas provides five analytic modules, including cell-type prediction, identification of marker genes, functional enrichment analysis, identification of cell-type-specific transcription regulons, and cell-cell communication analysis. In addition, users can upload scRNA-seq data for personalized analysis. CardioAtlas is available at http://bio-bigdata.hrbmu.edu.cn/CardioAtlas. CardioAtlas provides the first comprehensive and well-crafted reference atlas of cardiovascular systems and diseases and describes in detail previously unrecognized cell populations across a large number of humans and mice.

**Supplementary Information:**

The online version contains supplementary material available at 10.1186/s40364-024-00696-5.

*To the editor*.

Cardiovascular disease is the leading cause of morbidity and mortality worldwide. Exploring the cellular composition and ecosystem is crucial for resolving disease progression. In addition to a high proportion of myocardial cells, there are also many other types of cells in the heart, such as endothelial cells and fibroblasts (FBs) [1]. Different cell types play diverse roles in the development of cardiovascular diseases. The application of single-cell RNA sequencing (scRNA-seq) has intensified our understanding of cell states and heterogeneity of cardiovascular disease [2, 3]. Numerous studies have led to the accelerated accumulation of a large amount of scRNA-seq datasets of cardiovascular systems and various diseases. However, there are only a few data resource related to cardiovascular diseases, such as CVDHD, which was built ten years ago. There is still a lack of a specialized single-cell database for cardiovascular diseases and tissues.

Therefore, we developed CardioAtlas, a comprehensive scRNA-seq data resource and analysis platform to decipher cell composition of cardiovascular diseases (Fig. 1 and Fig. S1). The CardioAtlas includes 3,016,715 cells in 15 diseases and 2 tissues from published literature (Fig. 1A). Over 60 cell types were annotated based on canonical markers. We constructed the context-specific single cell reference atlas for cell type annotation for human and mouse cardiovascular diseases and tissues respectively, which were available for users to flexibly input into various cell type prediction algorithms (Fig. 1B-C). To facilitate researchers in reusing these datasets, we have provided five practical downstream analysis modules, including marker gene analysis, functional annotation, regulatory module analysis and cell-cell communication (Fig. 1D-I). The platform integrated functional modules such as query, browsing, and online tools (Fig. 1J-L). Results are visually displayed in various forms of graphs and tables. All scRNA-seq data, reference atlas, graphs, and analysis results are supported for download. Users can upload and select a reference atlas to analyze the scRNA-seq data online. The detailed usage was shown in Fig. S2.

**Fig. 1:**
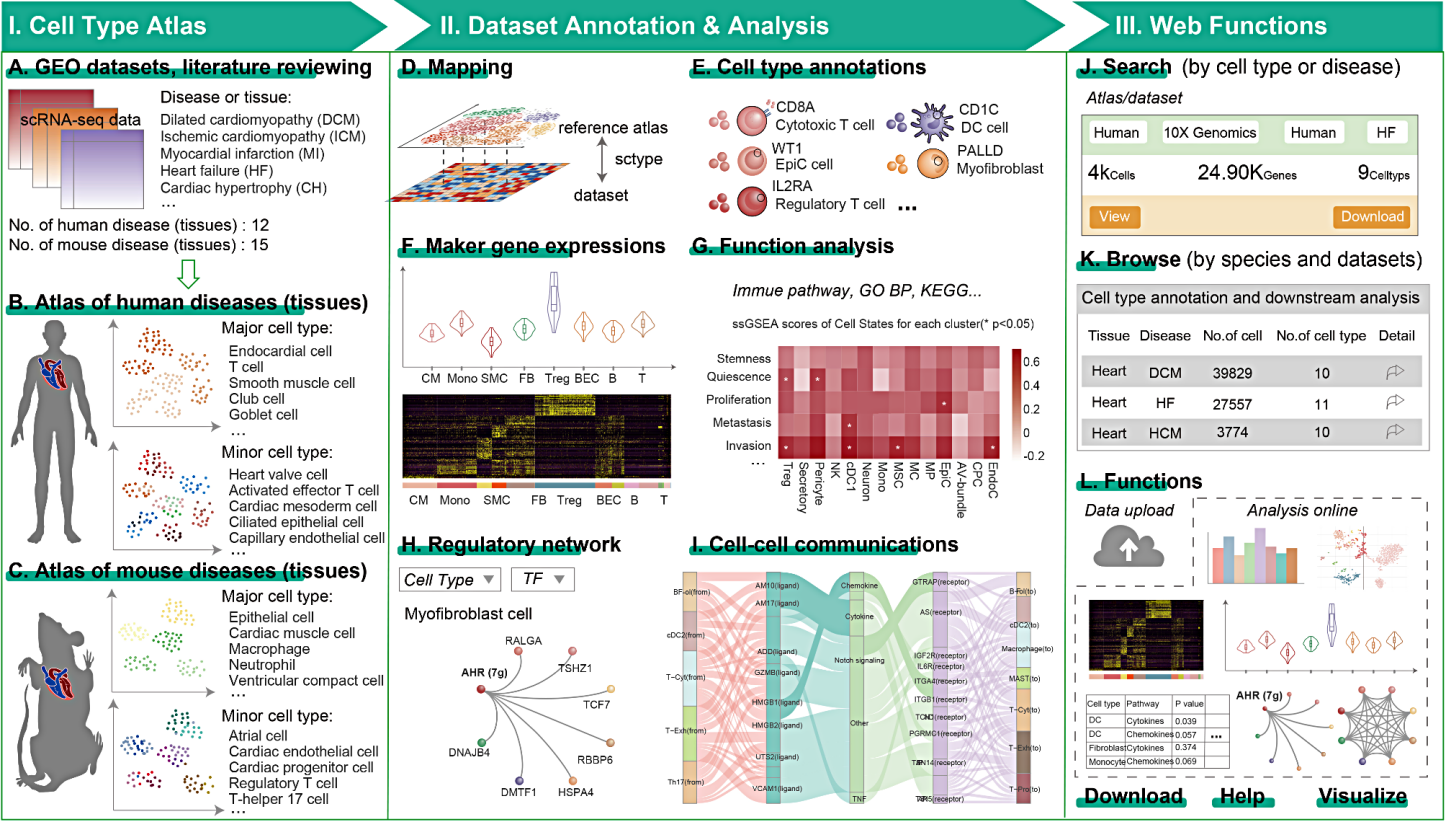
Schematic of overall design of CardioAtlas. (**A**) Collection of reference transcriptomes from human and mouse cardiovascular diseases and tissues from public studies. (**B**)-(**C**) scRNA-seq atlas of human and mouse cardiovascular diseases and tissues. (**D**)-(**E**) Computational methods for automatically annotate cell types. (**F**)-(**I**) Four main types of analysis provided in CardioAtlas. (**J**)-(**L**) Interface for CardioAtlas web functions

To demonstrate the application of CardioAtlas, we comprehensively analyzed the publicly available scRNA-seq datasets of cardiovascular diseases from a recent study [4]. The overall accuracy for annotating minor cell types from patients with hypertrophic cardiomyopathy (HCM) by CardioAtlas reached 0.945 (Fig. 2A-B). In addition, we found that POSTN, COL22A1, and DCN, which previously reported as marker genes of fibroblasts (Fig. 2C), were significantly overexpressed in FB cells [4, 5]. Functional analysis showed that most cell type specifically up-regulated genes were significantly enriched in the related process pathways (Fig. 2D). For example, genes highly expressed in FB cells, Neuron cells, and SMC cells were significantly enriched in the neurogenesis pathway, which were consistent with the previous study [4]. Based on the cell-cell communication analysis, it was found that THBS4-CD36 interaction facilitates communication between myofibroblasts (MFBs) and pericytes, cardiac muscle myoblast (CMMs), as well as fat cells (Fig. 2E). THBS4 belongs to the thrombospondin family, which could mediate extracellular matrix protein production and cellular attachment dynamics. Previous studies revealed overexpressed THBS1 induced lethal cardiac atrophy [6] and THBS4 could regulate vascular inflammation and atherogenesis [7]. CD36 was often considered as a receptor for THBS1 and could regulate the cAMP signaling pathway, which is a major second messenger in the heart [8, 9]. Therefore, the results here emphasize the important role of THBS4-CD36 in the development of HCM, providing a potential therapeutic target.

**Fig. 2:**
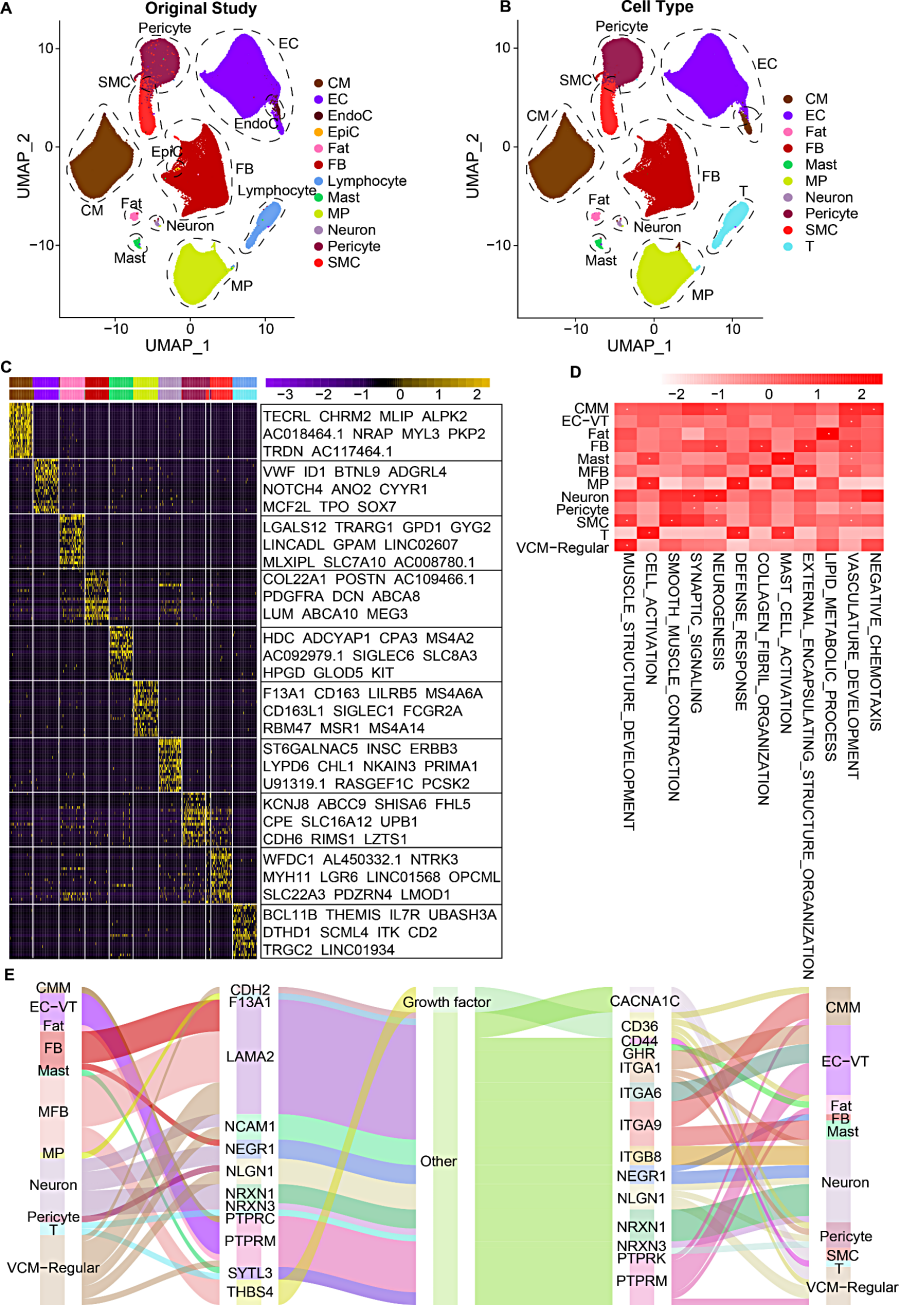
Case study of human HCM scRNA-seq data analysis based on CardioAtlas. (**A**) UMAP plot showing the cell annotations from original study. (**B**) UMAP plot showing the minor cell type annotations based on CardioAtlas. (**C**) Heat map showing the expressions of marker genes in various cell types. (**D**) Functional pathways enriched by genes highly expressed in cell types. *p < 0.05. (**E**) Cell-cell communications mediated by ligand-receptor pairs

Meanwhile, we also validated the accuracy of annotation of cell types and analysis results using an independent scRNA-seq data of mouse cardiovascular diseases [10]. CardioAtlas was applied to align minor cell types accurately, with an overall annotation accuracy of 0.922 (Fig. S3A-B). Next, we identified genes highly expressed in various cell types based on the analysis module in CardioAtlas (Fig. S3C). We found that most cell type specifically expressed genes have been validated by previous studies. For example, Ttn was highly expressed in CM cells, and Pdgfra was detected to be highly expressed in FB cells [10, 11]. In addition, we performed functional enrichment analysis based on CardioAtlas. We found that marker genes of several cell types, such as EC cells, FB cells, and T cells were significantly enriched in the cell adhesion pathway (Fig. S3D). Genes highly expressed in immune related cells such as MP cells and T cells were significantly enriched in the immune response pathway (Fig. S3D). Moreover, genes highly expressed in CM cells are significantly enriched in the muscle system process pathway, which is consistent with their functions reported in the previous study [10]. Finally, we analyzed the cell-cell communications mediated by various ligand-receptor interactions (Fig. S3E). The interaction between Cd74 and App facilitates communication between MP cells and other cell types, such as EC cells, FB cells, and T cells, consistent with previous research [12].

In conclusion, CardioAtlas is one of the most comprehensive scRNA-seq resource for human and mouse cardiovascular diseases and tissues. CardioAtlas provided context-specific reference atlas for cell type annotation and useful analysis results for users without complex operational processes. We believe that CardioAtlas will facilitate the dissecting of cellular composition and heterogeneity in complex cardiovascular disease and tissues.

## Electronic supplementary material

Below is the link to the electronic supplementary material.

Supplementary Material 1

Supplementary Material 2

## Data Availability

No datasets were generated or analysed during the current study.
