# Supplementary material for: CardioAtlas: deciphering the single-cell transcriptome landscape in cardiovascular tissues and diseases
Source: Biomark Res. 2024 Nov 28;12:149. doi: 10.1186/s40364-024-00696-5 (PMC11606023; doi:10.1186/s40364-024-00696-5)
Supplement: Supplementary file 1 — Supplementary Material 1 [file 40364_2024_696_MOESM1_ESM.pdf]

Methods

Collection and quality control of scRNA-seq data

We collected the single-cell sequencing data by querying Gene Expression Omnibus (GEO, https://www.ncbi.nlm.nih.gov/geo/)(1) and public literature with ‘cardiovascular’, ‘aorta’ and ‘heart’ as keywords. In total, we obtained 66 datasets (27 in human and 39 in mouse) from diverse sequencing platforms, including 10x Genomics, STRT-seq, sNucDrop-seq, ICELL8, and Drop-seq (Fig. S1). Gene expression matrix of each dataset were obtained and dealt with Seurat (V4.3)(2) to go through a quality control analysis.

For gene filtering, genes that were expressed in less than 3 cells were removed. For cell filtering, cells expressed with less than 200 genes were removed, and cells were selected following the principles of original literature. If principles not mentioned in the original literature, ‘isOutlier’ function from scater (V1.28) R package, which was based on median absolute deviations (MADs), was used to cell filtration. The outlier cells were defined in each of the following metrics and removed: log(UMI counts) (>2 MADs, both end), log(number of genes expressed) (>2 MADs, both end) and log(percent mitochondrial read count) (>2 MADs, high end). Next, scDblFinder (V1.14) R package was used to detect potential doublets in each sample. Finally, a total of 3,016,715 single-cell transcriptomes in human and mouse were retained after quality control (Fig. S1B).

Dimensionality reduction and clustering of scRNA-seq data

‘SCTransform’(3) was used to normalize, scale and identify variable genes for

scRNA-seq data. After normalization, we calculated cell-cycle scores for each cell based on the genes of S phase and G2M phase. By scaling, variations triggered by mitochondrial gene and cell cycle gene were removed. Next, we constructed a gene blacklist, including mitochondrial genes, ribosomal genes, immunoglobulin genes and proliferating genes. The top 1500 most variable genes were identified, excluding genes in the blacklist, for subsequent dimensional reduction and clustering analysis. Principal component analysis (PCA) was performed and the first 25 components were selected for further processing. The shared-nearest-neighbors graph was constructed based on the Euclidean distance in the low-dimensional subspace. The clusters were calculated by the FindClusters function with a resolution of 0.5 and visualized using the uniform manifold approximation and projection (UMAP) plots. After cluster analysis, differentially up-regulated genes were identified over all pairs of clusters.

Constructing the reference atlas of cell types

For the same disease or tissue, multiple datasets were integrated and scaled. The batch effects from different samples or datasets were removed by Harmony(4). A basic single-cell data processing is executed as described above for each integrated data. Then, marker genes were identified and clusters were annotated based on canonical marker genes collected manually. Then, we labeled cells with major cell types (e.g. T cell, endothelial cell) and minor cell types (e.g. regulatory T cell, Endothelial cell of high endothelial venule). Based on the annotated cell types, we constructed the disease- or tissue-specific reference profiles for human and mouse with a specific format of computational method ‘ScType’(5).

Cell type annotation for single dataset

For each scRNA-seq data, we annotated cell types for datasets based on the disease- or tissue-specific reference atlas. After the basic single-cell data processing, each scaled expression profile was input into ScType. Eventually, major cell types and minor cell types were identified for single dataset.

Cell type-specific expression of genes and functional assignments

Based on the annotations of cell types, we used COSine similarity-based marker Gene identification (COSG) to identify the cell type-specifically highly expressed genes when compared with other cell types. COSG is a cosine similarity-based method, which is faster than 'findAllMarkers' of Seurat. Top highly expressed 100 genes were selected for each cell type.

To investigate the functions of cell types, we first calculated the single sample gene set enrichment analysis (ssGSEA) scores for each cell. The cell states(6), immune signatures(7) and cancer hallmarks(8) were considered. In addition, we performed hypergeometric test to identify the significantly enriched functions based on highly expressed genes. The genes with FDR <0.01 and fold-changes > 2 were identified as highly expressed genes. Functions with $p < 0.05$ were identified as significant results.

Gene regulator network analysis

To explore the regulons of cell types, we applied pySCENIC to scRNA-seq expression profiles. The regulators and corresponding targets were reconstructed for each cell and the activity was assessed. Based on the cellular activity patterns, SCENIC identified meaningful clusters of cells and their potential regulons.

pySCENIC provides nonlinear projection methods to display visual groupings of cells based on the cellular activity patterns of these regulons and improve the speed remarkably.

Cell-Cell communications

To further explore the interactions between cell types of cardiovascular tissues and diseases, CardioAtlas integrated iTALK (https://github.com/Coolgenome/iTALK) to provide cell-cell communication analysis. The union sets of ligand-receptor pairs were integrated from CellchatDB(9), celltalkDB(10), ICELLNET(11), iTALK, Nichenet(12), singlecellsignalR(13) and one recent study(14).

Database implementation

The front-end of CardioAtlas is built with HTML5, JavaScript, and CSS code that consists of jQuery (v3.3.1), Datatable (1.10.25) ECharts (V5.5.1) and D3 (7.6.1) plugin. The back-end of CardioAtlas is powered by MySQL (v5.5.21) and queried via the Java Server Pages with Apache Tomcat container (v 6.0) as the middleware. All data in CardioAtlas were stored and managed using MySQL (version 5.5.21). Moreover, we employed Java and R program to perform online analyses. CardioAtlas has been tested on several popular web browsers, including Google Chrome, Firefox or Apple Safari browsers.

References

1. Barrett T, Wilhite SE, Ledoux P, Evangelista C, Kim IF, Tomashevsky M, et al. NCBI GEO: archive for functional genomics data sets--update. Nucleic Acids Res.

2013;41(Database issue):D991-5.

2. Butler A, Hoffman P, Smibert P, Papalexi E, Satija R. Integrating single-cell transcriptomic data across different conditions, technologies, and species. Nature biotechnology. 2018;36(5):411-20.

3. Hafemeister C, Satija R. Normalization and variance stabilization of single-cell RNA-seq data using regularized negative binomial regression. Genome biology. 2019;20(1):296.

4. Tran HTN, Ang KS, Chevrier M, Zhang X, Lee NYS, Goh M, et al. A benchmark of batch-effect correction methods for single-cell RNA sequencing data. Genome biology. 2020;21(1):12.

5. Ianevski A, Giri AK, Aittokallio T. Fully-automated and ultra-fast cell-type identification using specific marker combinations from single-cell transcriptomic data. Nat Commun. 2022;13(1):1246.

6. Yuan H, Yan M, Zhang G, Liu W, Deng C, Liao G, et al. CancerSEA: a cancer single-cell state atlas. Nucleic Acids Res. 2019;47(D1):D900-d8.

7. Li Y, Jiang T, Zhou W, Li J, Li X, Wang Q, et al. Pan-cancer characterization of immune-related lncRNAs identifies potential oncogenic biomarkers. Nat Commun. 2020;11(1):1000.

8. Liberzon A, Birger C, Thorvaldsdóttir H, Ghandi M, Mesirov JP, Tamayo P. The Molecular Signatures Database (MSigDB) hallmark gene set collection. Cell systems. 2015;1(6):417-25.

9. Jin S, Guerrero-Juarez CF, Zhang L, Chang I, Ramos R, Kuan CH, et al.

Inference and analysis of cell-cell communication using CellChat. Nat Commun. 2021;12(1):1088.

10. Shao X, Liao J, Li C, Lu X, Cheng J, Fan X. CellTalkDB: a manually curated database of ligand-receptor interactions in humans and mice. Briefings in bioinformatics. 2021;22(4).

11. Noël F, Massenet-Regad L, Carmi-Levy I, Cappuccio A, Grandclaudon M, Trichot C, et al. Dissection of intercellular communication using the transcriptome-based framework ICELLNET. Nat Commun. 2021;12(1):1089.

12. Browaeys R, Saelens W, Saeys Y. NicheNet: modeling intercellular communication by linking ligands to target genes. Nature methods. 2020;17(2):159-62.

13. Cabello-Aguilar S, Alame M, Kon-Sun-Tack F, Fau C, Lacroix M, Colinge J. SingleCellSignalR: inference of intercellular networks from single-cell transcriptomics. Nucleic Acids Res. 2020;48(10):e55.

14. Ramilowski JA, Goldberg T, Harshbarger J, Kloppmann E, Lizio M, Satagopam VP, et al. A draft network of ligand-receptor-mediated multicellular signalling in human. Nat Commun. 2015;6:7866.

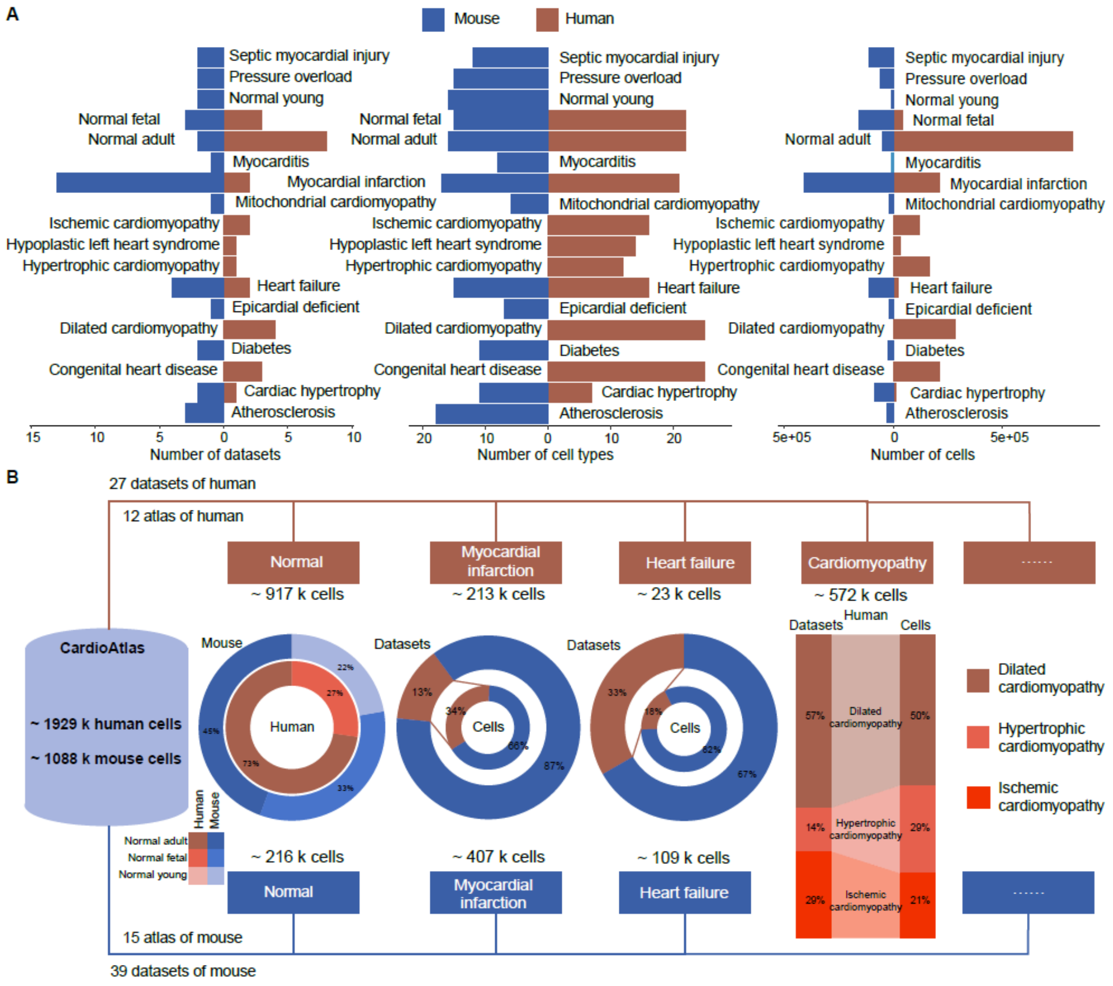

Figure S1. An overview of data collection for human and mouse cardiovascular diseases and tissues. (A) Barplots showing the number of datasets, cell types and cells in individual cardiovascular disease. (B) Pie charts and river plot showing the proportion of cells in each disease.

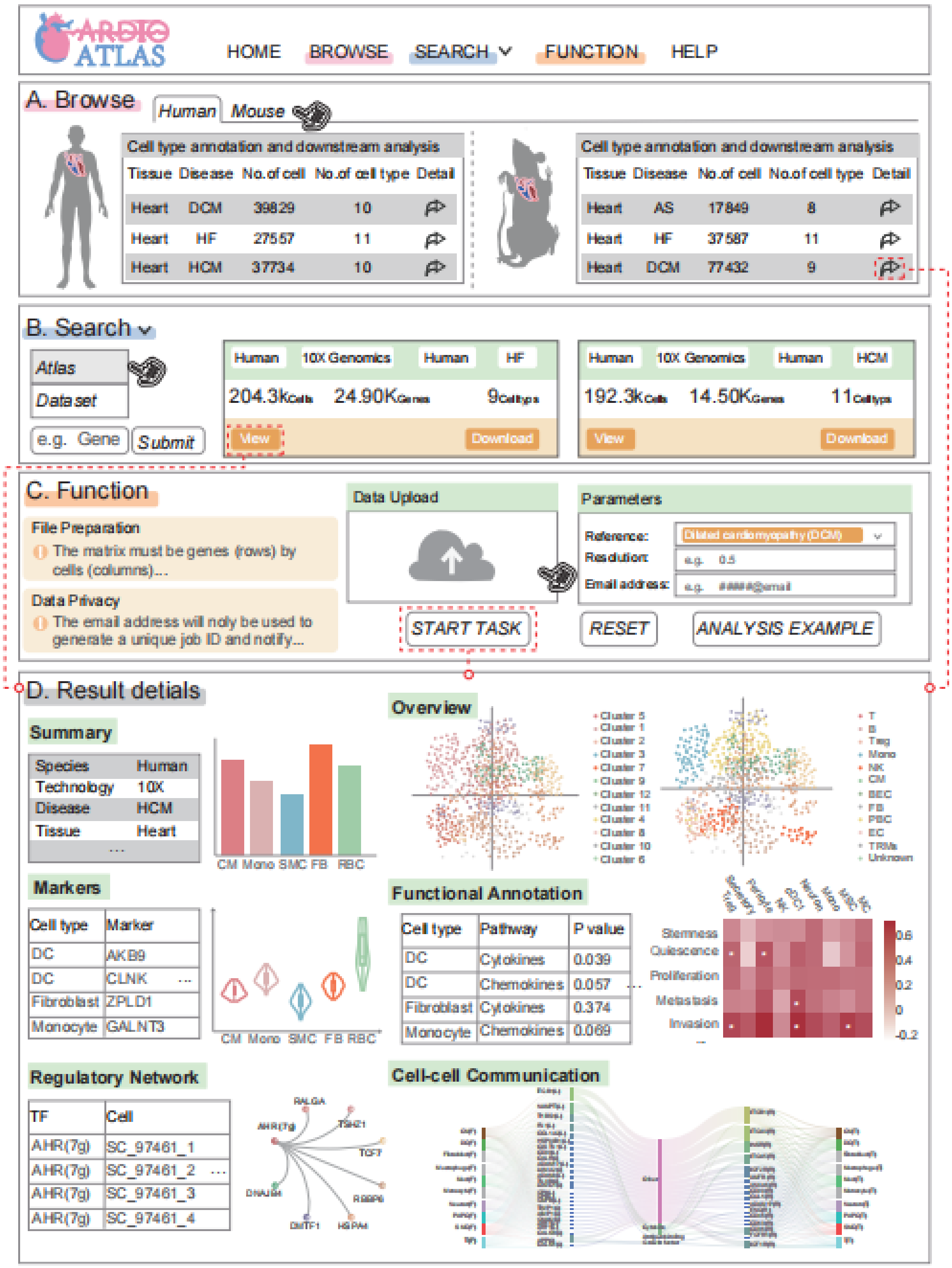

Figure S2. User interface and workflow of CardioAtlas. (A) The browse page of CardioAtlas. (B) The search page of CardioAtlas. (C) The function page of CardioAtlas. (D) Annotation results from browse, search, or function page, including summary of dataset, overview of cell type annotations, expressions of marker genes, function annotations, regulatory network analysis, and cell-cell communications.

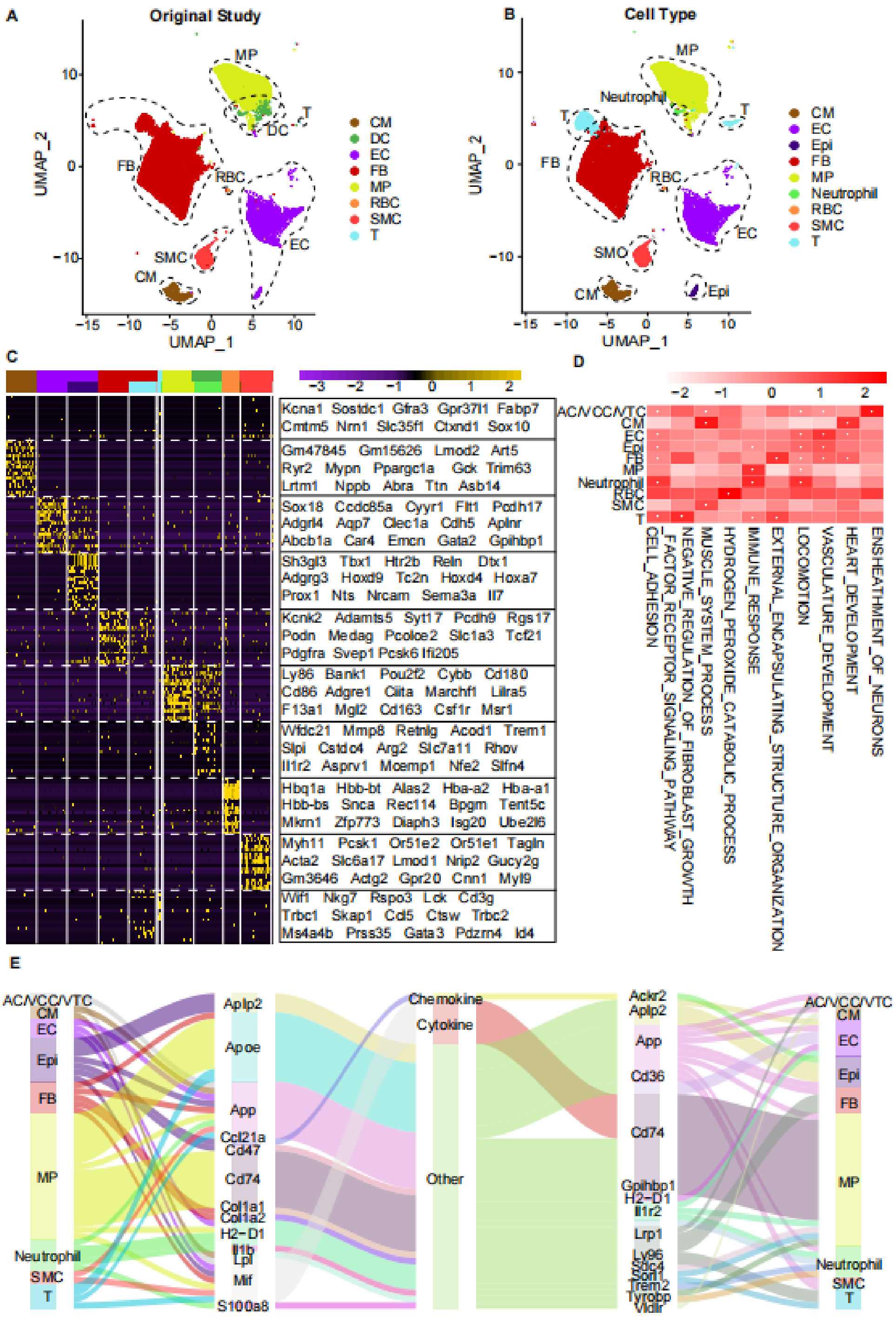

Figure S3. Case study of mus musculus cardiovascular disease scRNA-seq data analysis based on CardioAtlas. (A) UMAP plot showing the cell annotations from original study. (B) UMAP plot showing the minor cell type annotations based on CardioAtlas. (C) Heat map showing the expressions of marker genes in various cell types. (D) Functional pathways enriched by genes highly expressed in cell types. *p<0.05. (E) Cell-cell communications mediated by ligand-receptor pairs.
